# Overexpression Effects of miR-424 and BMP2 on the Osteogenesis of Wharton's Jelly-Derived Stem Cells

**DOI:** 10.1155/2021/7031492

**Published:** 2021-11-08

**Authors:** Asghar Fallah, Mahdieh Alipour, Zahra Jamali, Akbar Farjadfar, Leila Roshangar, Minoo Partovi Nasr, Parisa Hashemi, Marziyeh Aghazadeh

**Affiliations:** ^1^Iranian Institute of Cell and Gene Therapy, Tehran, Iran; ^2^Dental and Periodontal Research Center, Faculty of Dentistry, Tabriz University of Medical Sciences, Tabriz, Iran; ^3^Stem Cell Research Center, Tabriz University of Medical Sciences, Tabriz, Iran; ^4^Oral Medicine Department of Tabriz University of Medical Sciences, Tabriz, Iran; ^5^Department of Medical Biotechnology, Fasa University of Medical Sciences, Fasa, Iran; ^6^Faculty of Dentistry, Tabriz University of Medical Sciences, Tabriz, Iran

## Abstract

Recently, the translational application of noncoding RNAs is accelerated dramatically. In this regard, discovering therapeutic roles of microRNAs by developing synthetic RNA and vector-based RNA is attracting attention. Here, we studied the effect of BMP2 and miR-424 on the osteogenesis of Wharton's jelly-derived stem cells (WJSCs). For this purpose, human BMP2 and miR-424 DNA codes were cloned in the third generation of lentiviral vectors and then used for HEK-293T cell transfection. Lentiviral plasmids contained miR424, BMP-2, miR424-BMP2, green fluorescent protein (GFP) genes, and helper vectors. The recombinant lentiviral particles transduced the WJSCs, and the osteogenesis was evaluated by real-time PCR, Western blot, Alizarin Red staining, and alkaline phosphatase enzyme activity. According to the results, there was a significant increase in the expression of the BMP2 gene and secretion of Osteocalcin protein in the group of miR424-BMP2. Moreover, the amount of dye deposition in Alizarin Red staining and alkaline phosphatase activity was significantly higher in the mentioned group (*p* < 0.05). Thus, the current study results clarify the efficacy of gene therapy by miR424-BMP2 vectors for bone tissue engineering. These data could help guide the development of gene therapy-based protocols for bone tissue engineering.

## 1. Introduction

The skeleton microstructure is made up of mineralized extracellular matrix and bone remodeling units, including osteocytes, osteoblasts, osteoclasts, and lining cells. According to this complex structure and limitations in the self-healing ability of defected bones, reconstruction of bone tissue remains challenging [1, 2]. Autologous bone graft is the conventional therapeutic approach that has been known as the gold standard [3–6]. Despite the positive results, this method is limited by disadvantages such as morbidity of the donor site, shortage in blood supply, restrictions in the size of the graft, and fixation challenges [7]. There are successful repair reports for femoral, mandibular, and tibia defects by different mesenchymal stem cells stimulated by inductive factors [8–17], but a recent meta-analysis showed that their results are not as successful as autologous grafts [18].

For nearly half a century, gene therapy has attracted attention in regenerative medicine. Despite the problems, considerable improvement has been made in this field [19]. There are several approved, ongoing, or finished clinical trials based on gene therapy to treat critical medical problems such as cancer, lipase deficiency, blindness, Alzheimer's disease, and HIV disease [20]. Fully functional regenerated bone defects are an ultimate goal in bone tissue engineering, which could be promised by gene therapy-based methods [21].

Osteoblastic differentiation of stem cells is regulated by different genes that are coding several secretive molecules and transcription factors, including bone morphogenetic proteins (BMPs), Wnt proteins, Indian hedgehog homolog (IHH), and fibroblast growth factors (FGFs). These essential molecules activate different intracellular and extracellular pathways through autocrine or paracrine signals to regulate the expression of transcription factors [22].

BMP-2, a transforming growth factor-*β* superfamily subgroup, has a critical role in bone development and regeneration [23–25]. Recombinant human BMP-2 (rhBMP-2) and recombinant human BMP-7 (rhBMP-7) are the only approved subgroup for clinical application in bone fracture [26]. This substance has the same fusion rate and clinical outcomes as autologous iliac bone grafts [27]. The LT-CAGE is a commercial form of rhBMP-2 loaded in a collagen sponge in the USA, used for the clinical treatment of degenerative lumbar disc disease [26]. Several studies mentioned successful results of the controlled release of BMP2 in bone regeneration [2, 28–30]. Meanwhile, some drawbacks have been reported, including soft tissue swelling, cyst formation, implant migration, local inflammation, and, more importantly, increased risk of malignancy in high-dose administration [31]. Besides osteogenesis, this protein promotes angiogenesis by increasing microvessel formation in distinct concentrations [32]. This process plays a vital role in bone formation, fracture healing, and bone distraction [33–35].

The isolation and production processes of BMP2 protein are costly. Moreover, the rapid clearance of this factor through different mechanisms causes the necessity of multiple clinical injections or the application of sustained delivery systems [36]. So, it seems more convincing to transfer the responsible gene and gain sustained protein production in a physiological pattern [37].

A cluster of microRNAs (miRNAs) has been recognized to play an essential role in the entire process of stem cell differentiation. MicroRNAs are short (20-24 nt) noncoding RNAs (ncRNAs) that are involved in posttranscriptional regulation of gene expression in multicellular organisms [38, 39]. There is growing evidence about the crucial role of ncRNAs in epigenetic control and stem cell fate, including pluripotency maintenance and lineage-specific differentiation. Individually or together, miRNAs are involved in placenta, heart, skeletal, and muscle development during embryogenesis [40, 41]. Several studies reported the promotion or inhibition effects of different miRNAs in the bone formation process. The previous studies showed that miRNAs enhance the level of expression of osteogenic transcription factors, resulting in promoting osteogenic differentiation. Over a decade, a large body of knowledge has accumulated related to the H19X-encoded miR-424(322)/-503 cluster roles in stem cell differentiation and proliferation [42]. Recently, the role of this miRNA was evaluated in osteogenesis. According to these studies, upregulation of miR-322 (424), miR-34a, and miR10a expression follows by the overexpression of runt-related transcription factor-2 (Runx-2), osterix (Osx), and osteocalcin (OCN). These complicated signaling pathways regulate bone formation. Among hundreds of miRNAs controlling this process, few studies have analyzed the role of miR424 (MiR322). miR-322 is reported as a regulator in osteoblast differentiation. Overexpression of this microRNA enhances the BMP-2 response in bone marrow stem cells [43]. Due to the reports, the expression of osteogenic genes such as Osx, Runx2, Msx2, and Ibsp is increased by targeting Tob2 by miR-322 [43, 44]. However, there is limited information and some controversies about the effect of miR424 on osteoblast differentiation.

In this study, we mainly focused on the emerging role of miRNA 424 in enhancing the overexpression of the BMP2 gene followed by mineralization. This study provides more evidence for a combination of coding and noncoding gene therapy and helps develop innovative gene therapy studies using novel biomaterials and advanced bioprinting techniques in bone regeneration.

## 2. Materials and Methods

### 2.1. Ethical Issue

This experiment was an interventional in vitro study, approved at the Stem Cell Research Center of Tabriz Medical Science University, Iran, with the Ethical Code of IR.TBZMed.7CR.REC. 13970268.

### 2.2. Design and Synthesis of the Expression Construct

The BMP2 (NM_001200) and miR-424 (NR_029946) coding sequences were extracted from the NCBI databases and then were synthesized as a single open reading frame (ORF). The construct was synthesized and cloned into a pUC57 vector (General Biosystems, USA). To develop destination vectors including miR424, hBMP2, and hBMP2-miR424, the constructs were subcloned from the pUC57 vector into the pCDH513-B. The pCDH513-B lentiviral vector, which contained copaGFP and Puromycin (pCDH-GP) under EF-1 promoter, was used as the destination vector. A few clones were purified after confirming with colony PCR by applying the Miniprep Plasmid Kit (ThermoFisher, USA). The right clone that contained 300 ng of plasmid DNA was digested with restriction enzymes (XhoI, EcoRI, and BamHI) in a water bath at 37°C for 40 minutes, then run on agarose gel for analysis. Ultimate confirmation was done with sequencing.

### 2.3. Recombinant Lentivirus Production, Titration, and Concentration

Human embryonic kidney (HEK-293T) cells were used for lentivirus packaging. HEK-293T cell lines (ThermoFisher, USA) were cultured in 75 cell culture flasks in Dulbecco's modified Eagle's medium (D-MEM, Gibco, USA) containing 10% fetal bovine serum (FBS, Gibco, USA) and incubated at 37°C temperature with 5% CO_2_. When the HEK-293T cells reached about 60%-70% confluence, they were transfected for packaging recombinant lentivirus. Different tetratransfer vectors, pCDH-hBMP2-miR424-GP, pCDH-hBMP2-GP, pCDH-miR424-GP, and pCDH-GP, were used for producing recombinant lentiviruses.

The Trono Lab (EPFL, Switzerland) protocols were applied for the production of recombinant lentivirus (rLV) and titration [45, 46]. The HEK-293T cells were transfected by different tetratransfer (21 *μ*g), helper PAX (15 *μ*g), and pMD2-G (10.5 *μ*g) vectors using calcium phosphate (CaPo4). The transfection rate was monitored after 14-16 hours by observing GFP intensity under a fluorescence microscope (Labomed, USA). The supernatant containing the recombinant virus was collected three times in the next 72 hours.

The supernatant containing cell debris was removed after centrifuging (1000 × g, 4°C, and 5 min). The plate was incubated for at least 12 hours at 4°C to concentrate the recombinant viruses, then precipitated at 10% polyethylene glycol (PEG, Sigma, USA) and centrifuged (4°C, 10000 × g). Flow cytometry on both crude and concentrated viruses was titration of the recombinant viruses [47]. Different volumes (1000, 500, 100, 50, 20, and 0 *μ*l) of the fresh viruses were applied for transducing HEK-293T cells in a 12-well plate. Moreover, concentrated viruses were used in 100, 10, 0, 10-1, and 10-2 *μ*l volumes.

### 2.4. Transduction of WJSCs

WJSCs were isolated and characterized as our team's previous study [48]. The umbilical cords of four donors were isolated, expanded, and characterized. Wharton jelly stem cells were seeded in a T75 flask in the medium culture containing DMEM-F12 and 10% FBS. After the cells reached the confluency of 70%, the recombinant lentiviral particles containing hBMP2-miR424, hBMP2, miR424, and GFP genes were added to the medium. The spinfection protocol (1500 rpm for 1.5 hours) was applied for WJSC transduction with the amount of recombinant lentiviral particle multiplicity of infection (MOI) equal to 5. A fresh culture medium with 10% FBS was added; then, the plates were transferred to the incubator. After 72 hours from transduction of the cells, the number of transduced cells was primarily evaluated by fluorescence microscopy (Labomed, USA). The mammalian selection marker, Puromycin, was applied for the removal of nontransduced cells. The puromycin (2 *μ*g/ml) treatment continued for 3-5 days, with everyday Puromycin containing fresh medium replacement.

The transduced cell viability was evaluated by MTT assay. About 5 × 10^3^ cells were cultured per well in 96-well plates. After 24 hours, MTT reagents (Carlsbad, CA, USA) were added and incubated for 4 hours. With the addition of DMSO, the MTT reaction was terminated. MTT was quantified by using absorbance readings via the microplate reader (BioTek, USA).

For osteogenic differentiation assay, six-well plates were applied. WJSCs were cultured with an amount of 0.3 × 10^6^ cells per well for qRT-PCR and 1 × 10^5^ cells for ELISA, ALP, and Alizarin Red staining tests. Each test was replicated three times per sample. The osteogenic differentiation media (ThermoFisher, USA) were applied for differentiation. The osteogenic medium was replaced every 72 hours. The incubation continued for seven days for qRT-PCR and alkaline phosphatase tests and 21 days due to Alizarin Red S staining and ELISA tests.

### 2.5. Investigation of mRNA BMP-2 and miRNA424

According to the manufacturer's protocol, the total RNA was extracted after seven days of osteogenic differentiation using the mRNA extraction kit (Qiagen, Germany). Real-time PCR was accomplished using 0.5 *μ*g RNA with the SYBR Green Master Mix. Primers used for qPCR are hmiR424F: CCTTCATTGACTCCGAGGGG, hmiR424R: ACCTTCTACCTTCCCCACGA, hBMP2F: 5′ACTCGAAATTCCCCGTGACC3′, and hBMP2R: 5′GGACACAGCATGCCTTAGGA, and as a control, hU6 F: GCTTCGGCAGCACATATACTAAAAT and hU6 R: CGCTTCACGAATTTGCGTGTCAT for hmiR424 and hGAPDH F: GATTTGGTCGTATTGGGCGC hGAPDHR: AGTGATGGCATGGACTGTGG used for human BMP2. The data were presented as the ratio of mean threshold targeted human exogenous gene expression to human endogenous human U6 and GAPDH. Analysis of the data was carried out by REST software.

### 2.6. ELISA Analysis and Effect of miR-424 and BMP2 on the Expression of Osteocalcin (OCN)

Osteocalcin (OCN) protein is usually applied as an indicator marker for osteogenesis differentiation. The concentration of OCN protein was measured in cell culture using the human OCN quantity ELISA kit (ab270202, Abcam, UK).

The supernatants of differentiated cells in the groups were collected 21 days after osteogenic induction. These cells were centrifuged, and the supernatants were evaluated for amounts of secreted OCN protein according to the manufacturer's instruction by a standard ELISA reader (Stat Fax 2100, Awareness Technologies, USA).

### 2.7. Mineralization Induction and ALP Activity in WJSCs

For quantitative ALP assay, the cells were washed with phosphate-buffered saline (PBS), lysed, and then mixed with 0.5 ml p nitrophenol phosphate solution (Sigma) and incubated for 15 min. Then, 10 ml of 0.05 N NaOH was added, and the supernatants were evaluated for ALP activity by the measurement at 405 nm [49].

For Alizarin Red S staining, as described in our previous study [50], cells were fixed, and calcium deposition in the plate was further evaluated by 2% Alizarin Red liquid (pH: 4.1–4.3)(Sigma–Aldrich, Steinem, Germany). After three-time washes in dH_2_O to remove the unbound stain, samples were air-dried and photographed by a microscope. For quantification, ImageJ software 1.52n version was used.

### 2.8. Statistical Analysis

Statistical analysis was performed using GraphPad Prism 8 (San Diego, CA, USA). All data were collected from three independent experiments and presented as mean ± standard deviation (SD). Differences were considered significant at *p* < 0.05, determined using one-way ANOVA with Tukey post hoc tests.

## 3. Results

### 3.1. Construction of Lentiviral-Based Bicistronic

**Figure 1:**
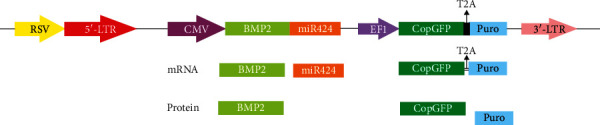
Plasmid construction: third-generation lentiviral vector was used. In this construct, expression of the BMP2-mir424 and copGFP-Puromycin was controlled under pCMV and eEF1 promoters, respectively. The copGFP gene was linked with the T2A peptide to Puromycin-resistant gene. The transfer vector generated two transcripts, and then, a miR and three proteins, BMP2, copGFP, and the puromycin-resistant marker, were made. The copGFP and the puromycin-resistant markers were used for monitoring and selection in transfection and transduction steps.

The three bicistronic vectors (System Bioscience, USA) containing BMP-2(pCDH-BMP2-GP), miR424 (pCDH-miR424-GP), and BMP-2-miR424 (pCDH-BMP-2-miR424-GP) sequences were constructed and confirmed by digestion and subsequent sequencing. The synthesized BMP2-miR424 genes were cloned under a strong human cytomegalovirus promoter (CMV). The copaGFP was linked with T2A peptide to Puromycin under the control of the EF1 promoter (Figure 1). The pCDH513-B empty lentiviral vector served as the control group (pCDH-GP).

As shown in this schematic, different versions of this vector were used, containing miR424 (pCDH-miR424-GP), hBMP-2 (pCDH-BMP2-GP), and BMP2 and miR424 (pCDH-BMP-2-miR424-GP). The pCDH513-B empty lentiviral vector served as control (pCDH-GP).

### 3.2. WJSC Transduction and Cell Proliferation Assay

**Figure 2:**
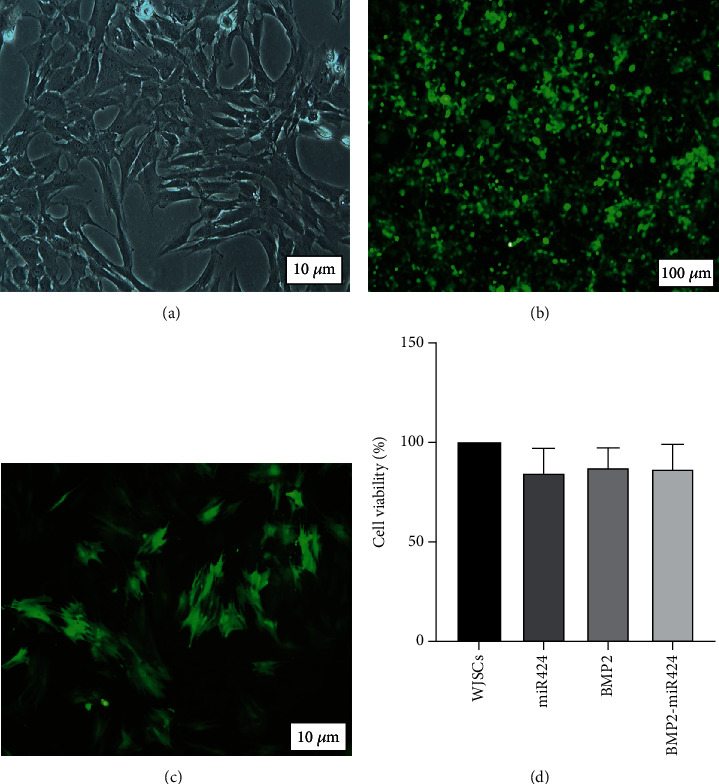
Transduction of WJSCs. (a) Morphology of isolated WJSCs cultured in DMEM-F12. (b) HEK293T cells in the packaging stage, secreting the virus-containing miR-424 and BMP2 gene. The green marker indicates the cells producing the virus. (c) WJSCs transduced by the virus. The green marker indicates cells infected with the virus and thus the genes integrated into the genome of the host cells. (d) Cell viability test. The viability of WJSCs was evaluated by MTT assay in the presence of miR424, BMP2, and BMP2-miR424 plasmids 72 hours after transduction. The differences were not statistically significant (*p* > 0.05).

According to the presence of GFP-positive cells, the transfection rate was 95-97% (Figure 2(a)). FACS titration showed 2.5 − 3 × 10^6^ recombinant particles in the supernatant culture medium, which reached 2.3 − 2.5 × 10^8^ particles/ml after PEG precipitation.

**Figure 3:**
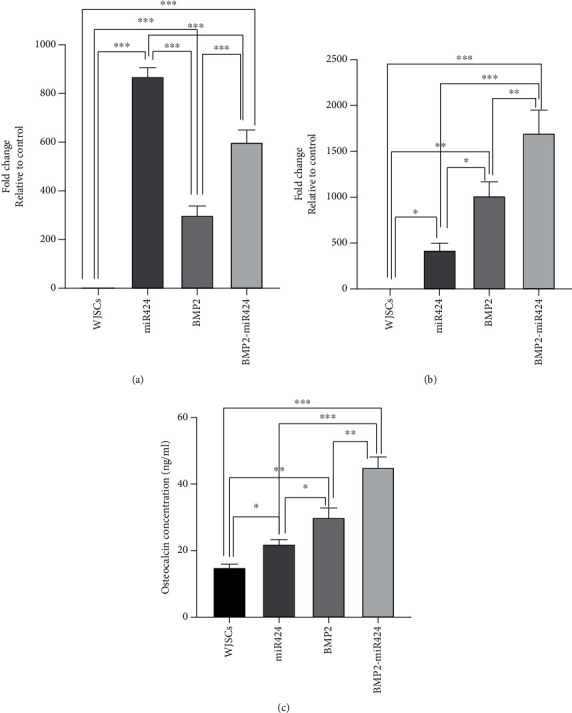
Quantitative polymerase chain reaction and ELISA analysis of Wharton's jelly stem cells. (a) miR424 gene expression was increased in miR424, BMP2, and BMP2-miR424 870.41, 300.16, and 600.36 folds, respectively. (b) Moreover, BMP2 gene expression elevated in all studied groups (miR424: 423.25, BMP2: 1015.35, and BMP2-miR424: 1700.11). (c) According to the results of ELISA analysis, the amount of Osteocalcin in the culture medium was alternately increased in studied groups. As shown in this figure, these amounts were increased in miR424, BMP2, and BMP2-miR424 groups. This increase was the highest in the BMP2-miR424 group. (^∗^*p* < 0.05, ^∗∗^*p* < 0.01, and ^∗∗∗^*p* < 0.001).

WJSCs were confirmed based on morphology (Figure 2(a)) and previous characterization process [48]. Cells were transduced at a MOI of 5 (Figure 3(b)). After 72 hours of transduction, the transduced cells were monitored by fluorescence microscopy. Recombinant cells were screened by puromycin antibiotic marker selection to remove not infected cells.

MTT analysis showed the viability of WJSCs in studied groups (Figure 2(d)). The percent of cell viability was 100%, 99.8%, 99.7%, and 99.6% in control, miR424, BMP2, and BMP2-miR424, respectively. However, among different groups, there were not any significant differences (*p* > 0.05).

### 3.3. Quantitative Polymerase Chain Reaction and ELISA Analysis

The qPCR was performed to evaluate the overexpression of miR-424 and BMP2. The endogenous U6 and GAPDH genes were selected as control. The BMP2 gene was significantly overexpressed in transduced WJSCs with the virus-containing miR424-BMP2 genes (Figures 3(a) and 3(b)). This difference was significant compared to WJSCs transduced with the virus-containing BMP2 or miR424 only (*p* < 0.05).

According to ELISA results, the amount of secreted OCN was also increased significantly in the BMP2-miR424 group compared to the other groups (Figure 3(c)).

### 3.4. Alkaline Phosphatase Activity and Alizarin Red S Staining

**Figure 4:**
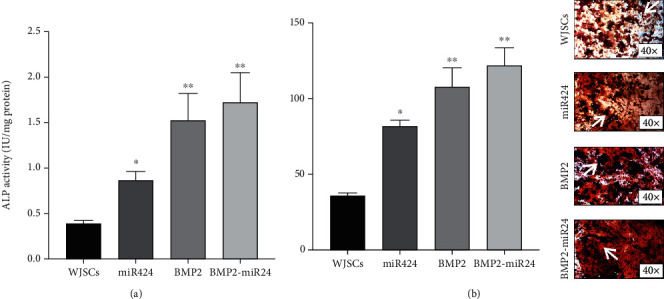
(a) Alkaline phosphatase activity of transduced cells and the control group. After seven days, the activity of this enzyme was significantly increased. The most activity was reported in the BMP2-miR424 group. (b) Alizarin Red images' analysis reported an increase of mineral deposition density in studied groups after 21 days. The red spots were shown by arrows. ^∗^*p* < 0.05; ^∗∗^*p* < 0.01.

According to the results of Alizarin Red staining and alkaline phosphatase (ALP) activity, osteoblastic differentiation and mineralization induction in WJSCs were elevated after exposure to study groups (Figure 4).

The ALP activity in all studied groups was significantly higher than that in the control group (*p* < 0.05) (Figure 4(a)).

The results of mineralization induction are shown in Figure 4(b). After evaluating by ImageJ software, the intensity of red color showed increased amounts of calcified nodules inBMP2-miR424, BMP2, and miR424 groups compared to the control group (*p* < 0.05). In conclusion, these results demonstrate that lentiviral-miR424-BMP2 transduction enhanced the osteogenic activity of WJSCs more than other studied groups.

## 4. Discussion

Bone tissue has a complex signaling network, and its efficient reconstruction needs full employment of growth factors and regulated genes, both coding and noncoding ones [51–53]. In the present study, we demonstrated that an *ex vivo* combination of coding gene and microRNA gene therapy increased the rate of osteogenesis. The application of our results of miR424 along with the BMP2 gene led to significant osteogenic promotion in WJSCs, which included upregulation of the OCN protein secretion and the mineral deposition in the transduced cells. In other words, this study cleared the positive effect of miR424 on the osteogenesis of mesenchymal stem cells.

Several miRNAs negatively or positively regulate the signaling of bone morphogenic proteins and vice versa. These proteins coordinate changes in miRNA expression. A study conducted by Li *et al.* showed an increase *in* osteogenic differentiation of C2C12 cells by downregulation of miR-135 [50, 54]. In another study, the increased expression of miR-206 inhibited the osteogenic differentiation in C2C12 cells [55]. Furthermore, it has been claimed that during osteogenic differentiation, the regulation of miR-218 was increased, and its expression enhanced osteoblastic markers, including Alp, Runx2, and OCN [56]. The mentioned studies highlighted the role of BMPs in the signaling pathways. Luzi *et al.* evaluated the contribution of microRNA 26a in osteogenic differentiation of human adipose tissue-derived stem cells (hASCs) and showed this differentiation is regulated through targeting the SMAD1 transcription factor, which is a signal transducer of BMPs [57]. Also, Mizuno *et al.* demonstrated the enhanced osteogenic induction in bone marrow-derived stromal cells by using miR-210 by targeting the *AcvR1b* receptor, a well-known member of the BMP receptor family [58]. An *in vivo* study demonstrated that the suppression of miR-2861 decreased the Runx2 protein expression, followed by a reduction of bone mass and density [59].

Previous *in vivo* and other *in vitro* studies showed that miR-424 is involved in regulating osteogenic differentiation and bone formation [60–62]. However, the exact mechanism and role are not thoroughly characterized. It has been shown that the expression of this microRNA is upregulated during osteogenic differentiation of bone marrow mesenchymal stem cells [63]. Meanwhile, there are some controversial studies in this regard. In a study, downregulation of miR-424 resulted in bone formation under oxidative stress. According to the results, the overexpression of miR-424 suppressed the proliferation and osteogenic differentiation in human adipose-derived mesenchymal stem cells. There was a decrease in alkaline phosphatase (ALP) activity and expression of osteogenic markers. However, this study was held under oxidative stress and claimed that the influence of miR-424 on osteogenesis is altered under this condition [60].

Moreover, it seems that the level of expression of miR-424 is variable during the osteogenesis process. The expression of this microRNA was higher in human bone marrow-derived mesenchymal stem cells (hBMSCs) than in osteoblasts [64]. In addition, an investigation using microarrays reported decreased miR-424 expression in osteogenically differentiated bone marrow stem cells [65, 66]. These results suggested that noncoding RNAs such as miR-424 play a critical role in maintaining bone growth and development at different development stages; meanwhile, their levels are markedly altered during osteogenesis.

To achieve the goal of bone regeneration, microRNA-424 seems to play an important role as triggers for overexpression of the BMP2 gene and proteins, which leads to increased mineralization. The addition of the BMP2 gene in the current study showed increased levels of the BMP2 gene expression besides enhanced mineralization induction and ALP activity [67]. There are similarities between this study and those described by Oishi *et al.* that evaluated the mineralization of PDGFRa+ cells in the presence of different microRNAs. Their study showed a notable decrease in the matrix mineralization of these cells after the suppression of miR-424 [68].

Moreover, in the beginning phase of matrix mineralization, the alkaline phosphatase enzyme is upregulated. The activity of this enzyme in the current study was upregulated notably in the miR424/BMP2 group.

Osteocalcin is a late marker of osteogenic differentiation and is expressed by osteoblastic-phenotype cells. This protein is mainly known as an abundant noncollagenous protein in the extracellular matrix of bone tissue [69]. In the current study, ELISA analysis of this protein showed significantly higher expression in WJSCs induced by miR424-BMP2 vectors.

The findings of our study suggest that induction of WJSCs by miR424-BMP2 leads to the upregulation of well-known osteoblastic factors. However, it seems that the determination of miR-424 cell signaling effect and *in vivo* studies about the effect of this noncoding RNA on osteogenesis are prerequired to clinical application of miR424-BMP2 vectors in the future.

## 5. Conclusion

Currently, the main biopharma industry products are based on recombinant protein. There are many pieces of evidence that noncoding RNAs play an essential role in human health. For the big part of noncoding RNA function in cell regulation and differentiation, applying noncoding genes as part of biotherapeutics agents is needed. The current study results clarify the efficacy of gene therapy by miR424-BMP2 vectors for bone tissue engineering. These data could help in guidance of the development of gene therapy-based protocols for bone tissue engineering.

## Data Availability

The data that support the findings of this study are available from the corresponding author, Dr. Aghazadeh, upon reasonable request.

## References

[1] Samiei M., Agazadeh M., Alizadeh E. (2016). Osteogenic/odontogenic bioengineering with co-administration of simvastatin and hydroxyapatite on poly caprolactone based nanofibrous scaffold. *Advanced Pharmaceutical Bulletin*.

[2] Schorn L., Sproll C., Ommerborn M., Naujoks C., Kübler N. R., Depprich R. (2017). Vertical bone regeneration using rhBMP-2 and VEGF. *Head & Face Medicine*.

[3] Schuckert K.-H., Jopp S., Osadnik M. (2010). Modern bone regeneration instead of bone transplantation: a combination of recombinant human bone morphogenetic protein-2 and platelet-rich plasma for the vertical augmentation of the maxillary bone—a single case report. *Tissue Engineering Part C: Methods*.

[4] Meyer U., Meyer T., Handschel J., Wiesmann H. P. (2009). *Fundamentals of tissue engineering and regenerative medicine*.

[5] Keestra J. A. J., Barry O., Jong L. ., Wahl G. (2016). Long-term effects of vertical bone augmentation: a systematic review. *Journal of Applied Oral Science*.

[6] Kahnberg K.-E., Nyström E., Bartholdsson L. (1989). Combined use of bone grafts and Brånemark fixtures in the treatment of severely resorbed maxillae. *International Journal of Oral & Maxillofacial Implants*.

[7] Fahmy R. A., Mahmoud N., Soliman S., Nouh S. R., Cunningham L., el-Ghannam A. (2015). Acceleration of alveolar ridge augmentation using a low dose of recombinant human bone morphogenetic protein-2 loaded on a resorbable bioactive ceramic. *Journal of Oral and Maxillofacial Surgery*.

[8] Bruder S. P., Kraus K. H., Goldberg V. M., Kadiyala S. (1998). The effect of implants loaded with autologous mesenchymal stem cells on the healing of canine segmental bone defects. *JBJS*.

[9] Dallari D., Fini M., Stagni C. (2006). In vivo study on the healing of bone defects treated with bone marrow stromal cells, platelet-rich plasma, and freeze-dried bone allografts, alone and in combination. *Journal of Orthopaedic Research*.

[10] Ito K., Yamada Y., Naiki T., Ueda M. (2006). Simultaneous implant placement and bone regeneration around dental implants using tissue-engineered bone with fibrin glue, mesenchymal stem cells and platelet-rich plasma. *Clinical Oral Implants Research*.

[11] Yuan J., Zhang W. J., Liu G. (2010). Repair of canine mandibular bone defects with bone marrow stromal cells and coral. *Tissue Engineering Part A*.

[12] Zheng Y., Liu Y., Zhang C. M. (2009). Stem cells from deciduous tooth repair mandibular defect in swine. *Journal of Dental Research*.

[13] Kon E., Muraglia A., Corsi A. (2000). Autologous bone marrow stromal cells loaded onto porous hydroxyapatite ceramic accelerate bone repair in critical-size defects of sheep long bones. *Journal of Biomedical Materials Research*.

[14] Berner A., Reichert J. C., Woodruff M. A. (2013). Autologous vs. allogenic mesenchymal progenitor cells for the reconstruction of critical sized segmental tibial bone defects in aged sheep. *Acta Biomaterialia*.

[15] Lee S.-H., Shin H. (2007). Matrices and scaffolds for delivery of bioactive molecules in bone and cartilage tissue engineering. *Advanced Drug Delivery Reviews*.

[16] Jeon O., Song S. J., Yang H. S. (2008). Long-term delivery enhances _in vivo_ osteogenic efficacy of bone morphogenetic protein-2 compared to short-term delivery. *Biochemical and Biophysical Research Communications*.

[17] Tabata Y. (2006). Regenerative inductive therapy based on DDS technology of protein and gene. *Journal of Drug Targeting*.

[18] Troeltzsch M., Troeltzsch M., Kauffmann P. (2016). Clinical efficacy of grafting materials in alveolar ridge augmentation: a systematic review. *Journal of Cranio-Maxillofacial Surgery*.

[19] Cox D. B. T., Platt R. J., Zhang F. (2015). Therapeutic genome editing: prospects and challenges. *Nature Medicine*.

[20] Shahryari A., Saghaeian Jazi M., Mohammadi S. (2019). Development and clinical translation of approved gene therapy products for genetic disorders. *Frontiers in Genetics*.

[21] Kim Y.-D., Pofali P., Park T. E. (2016). Gene therapy for bone tissue engineering. *Tissue Engineering and Regenerative Medicine*.

[22] Lin G. L., Hankenson K. D. (2011). Integration of BMP, Wnt, and notch signaling pathways in osteoblast differentiation. *Journal of Cellular Biochemistry*.

[23] Bandyopadhyay A., Tsuji K., Cox K., Harfe B. D., Rosen V., Tabin C. J. (2006). Genetic analysis of the roles of BMP2, BMP4, and BMP7 in limb patterning and skeletogenesis. *PLoS Genetics*.

[24] Shu B., Zhang M., Xie R. (2011). BMP2, but not BMP4, is crucial for chondrocyte proliferation and maturation during endochondral bone development. *Journal of Cell Science*.

[25] Tsuji K., Bandyopadhyay A., Harfe B. D. (2006). BMP2 activity, although dispensable for bone formation, is required for the initiation of fracture healing. *Nature Genetics*.

[26] Khan S. N., Lane J. M. (2004). The use of recombinant human bone morphogenetic protein-2 (rhBMP-2) in orthopaedic applications. *Expert Opinion on Biological Therapy*.

[27] McKay B., Sandhu H. S. (2002). Use of recombinant human bone morphogenetic protein-2 in spinal fusion applications. *Spine*.

[28] Kempen D. H., Lu L., Heijink A. (2009). Effect of local sequential VEGF and BMP-2 delivery on ectopic and orthotopic bone regeneration. *Biomaterials*.

[29] Mumcuoglu D., Fahmy-Garcia S., Ridwan Y. (2018). Injectable BMP-2 delivery system based on collagen-derived microspheres and alginate induced bone formation in a time- and dose-dependent manner. *European Cells & Materials*.

[30] Patel Z. S., Young S., Tabata Y., Jansen J. A., Wong M. E. K., Mikos A. G. (2008). Dual delivery of an angiogenic and an osteogenic growth factor for bone regeneration in a critical size defect model. *Bone*.

[31] Carragee E. J., Mitsunaga K. A., Hurwitz E. L., Scuderi G. J. (2011). Retrograde ejaculation after anterior lumbar interbody fusion using rhBMP-2: a cohort controlled study. *The Spine Journal*.

[32] Chen W.-C., Chung C. H., Lu Y. C. (2018). BMP-2 induces angiogenesis by provoking integrin *α*6 expression in human endothelial progenitor cells. *Biochemical Pharmacology*.

[33] Maes C., Carmeliet G., Schipani E. (2012). Hypoxia-driven pathways in bone development, regeneration and disease. *Nature Reviews Rheumatology*.

[34] Araldi E., Schipani E. (2010). Hypoxia, HIFs and bone development. *Bone*.

[35] Jacobsen K. A., al-Aql Z. S., Wan C. (2008). Bone formation during distraction osteogenesis is dependent on both VEGFR1 and VEGFR2 signaling. *Journal of Bone and Mineral Research*.

[36] Chen B., Lin H., Wang J. (2007). Homogeneous osteogenesis and bone regeneration by demineralized bone matrix loading with collagen-targeting bone morphogenetic protein-2. *Biomaterials*.

[37] Egermann M., Lill C. A., Griesbeck K. (2006). Effect of BMP-2 gene transfer on bone healing in sheep. *Gene Therapy*.

[38] MacFarlane L.-A., Murphy P. R. (2010). MicroRNA: biogenesis, function and role in cancer. *Current Genomics*.

[39] Krongbaramee T., Zhu M., Qian Q. (2021). Plasmid encoding microRNA-200c ameliorates periodontitis and systemic inflammation in obese mice. *Molecular Therapy-Nucleic Acids*.

[40] Gross N., Kropp J., Khatib H. (2017). MicroRNA signaling in embryo development. *Biology*.

[41] Huang T. H., Zhu M. J., Li X. Y., Zhao S. H. (2008). Discovery of porcine microRNAs and profiling from skeletal muscle tissues during development. *PLoS One*.

[42] Papaioannou G., Mirzamohammadi F., Kobayashi T. (2014). MicroRNAs involved in bone formation. *Cellular and Molecular Life Sciences*.

[43] Fröhlich L. F. (2019). Micrornas at the interface between osteogenesis and angiogenesis as targets for bone regeneration. *Cell*.

[44] Gámez B., Rodríguez-Carballo E., Bartrons R., Rosa J. L., Ventura F. (2013). MicroRNA-322 (miR-322) and Its Target Protein Tob2 Modulate Osterix (Osx) mRNA Stability. *Journal of Biological Chemistry*.

[45] Fallah A., Heidari H. R., Bradaran B., Sisakht M. M., Zeinali S., Molavi O. (2019). A gene-based anti-angiogenesis therapy as a novel strategy for cancer treatment. *Life Sciences*.

[46] Barde I., Salmon P., Trono D. (2010). Production and titration of lentiviral vectors. *Current Protocols in Neuroscience*.

[47] Szulc J., Wiznerowicz M., Sauvain M. O., Trono D., Aebischer P. (2006). A versatile tool for conditional gene expression and knockdown. *Nature Methods*.

[48] Hosseini A., Estiri H., Akhavan Niaki H. (2017). Multiple Sclerosis Gene Therapy with Recombinant Viral Vectors: Overexpression of IL-4, Leukemia Inhibitory Factor, and IL-10 in Wharton's Jelly Stem Cells Used in EAE Mice Model. *Cell Journal*.

[49] Alipour M., Firouzi N., Aghazadeh Z. (2021). The osteogenic differentiation of human dental pulp stem cells in alginate-gelatin/Nano-hydroxyapatite microcapsules. *BMC Biotechnology*.

[50] Inose H., Ochi H., Kimura A. (2009). A microRNA regulatory mechanism of osteoblast differentiation. *Proceedings of the National Academy of Sciences*.

[51] Peng S., Cao L., He S. (2018). An overview of long noncoding RNAs involved in bone regeneration from mesenchymal stem cells. *Stem Cells International*.

[52] Zhou Z., Hossain M. S., Liu D. (2021). Involvement of the long noncoding RNA H19 in osteogenic differentiation and bone regeneration. *Stem Cell Research & Therapy*.

[53] Hong L., Sun H., Amendt B. A. (2021). MicroRNA function in craniofacial bone formation, regeneration and repair. *Bone*.

[54] Li Z., Hassan M. Q., Volinia S. (2008). A microRNA signature for a BMP2-induced osteoblast lineage commitment program. *Proceedings of the National Academy of Sciences*.

[55] Sato M. M., Nashimoto M., Katagiri T., Yawaka Y., Tamura M. (2009). Bone morphogenetic protein-2 down-regulates miR-206 expression by blocking its maturation process. *Biochemical and Biophysical Research Communications*.

[56] Hassan M. Q., Maeda Y., Taipaleenmaki H. (2012). miR-218 Directs a Wnt Signaling Circuit to Promote Differentiation of Osteoblasts and Osteomimicry of Metastatic Cancer Cells∗. *Journal of Biological Chemistry*.

[57] Luzi E., Marini F., Sala S. C., Tognarini I., Galli G., Brandi M. L. (2008). Osteogenic differentiation of human adipose tissue-derived stem cells is modulated by the miR-26a targeting of the SMAD1 transcription factor. *Journal of Bone and Mineral Research*.

[58] Mizuno Y., Tokuzawa Y., Ninomiya Y. (2009). miR-210 promotes osteoblastic differentiation through inhibition of AcvR1b. *FEBS Letters*.

[59] Li H., Xie H., Liu W. (2009). A novel microRNA targeting HDAC5 regulates osteoblast differentiation in mice and contributes to primary osteoporosis in humans. *The Journal of Clinical Investigation*.

[60] Li L., Qi Q., Luo J. (2017). FOXO1-suppressed miR-424 regulates the proliferation and osteogenic differentiation of MSCs by targeting FGF2 under oxidative stress. *Scientific Reports*.

[61] Zhao W., Zhang S., Wang B., Huang J., Lu W. W., Chen D. (2016). Runx2 and microRNA regulation in bone and cartilage diseases. *Annals of the New York Academy of Sciences*.

[62] Hata A., Kang H. (2015). Functions of the bone morphogenetic protein signaling pathway through microRNAs (Review). *International Journal of Molecular Medicine*.

[63] Kim K. M., Lim S.-K. (2014). Role of miRNAs in bone and their potential as therapeutic targets. *Current Opinion in Pharmacology*.

[64] Gao J., Han J., Zhu H., Yang T., Fan Q., Baoan M. (2010). Expressions of miR-424 during differentiation of human bone marrow-derived mesenchymal stem cells. *Chinese Journal of Trauma.*.

[65] Vimalraj S., Selvamurugan N. (2014). MicroRNAs expression and their regulatory networks during mesenchymal stem cells differentiation toward osteoblasts. *International Journal of Biological Macromolecules*.

[66] Gao J., Yang T., Han J. (2011). MicroRNA expression during osteogenic differentiation of human multipotent mesenchymal stromal cells from bone marrow. *Journal of Cellular Biochemistry*.

[67] Yoshida Y., Tanaka S., Umemori H. (2000). Negative regulation of BMP/Smad signaling by Tob in osteoblasts. *Cell*.

[68] Oishi T., Uezumi A., Kanaji A. (2013). Osteogenic differentiation capacity of human skeletal muscle-derived progenitor cells. *PLoS One*.

[69] Tsao Y.-T., Huang Y. J., Wu H. H., Liu Y. A., Liu Y. S., Lee O. (2017). Osteocalcin mediates biomineralization during osteogenic maturation in human mesenchymal stromal cells. *International Journal of Molecular Sciences*.

